# Prospective quantitative gene expression analysis of kallikrein-related peptidase *KLK10* as a diagnostic biomarker for childhood acute lymphoblastic leukemia

**DOI:** 10.7717/peerj.13489

**Published:** 2022-05-31

**Authors:** Shwan Majid Ahmad, Basima Sadq Ahmed, Karzan Ghafur Khidhir, Heshu Sulaiman Rahman

**Affiliations:** 1 Department of Biochemistry, College of Medicine, University of Sulaimani, Sulaimaniyah, Iraq; 2 Department of Biochemistry & Clinical Chemistry, College of Pharmacy, University of Sulaimani, Sulaimaniyah, Iraq; 3 Department of Biology, College of Science, University of Sulaimani, Sulaimaniyah, Iraq; 4 Department of Physiology, College of Medicine, University of Sulaimani, Sulaimaniyah, Iraq

**Keywords:** Acute lymphoblastic leukemia, Chemotherapy, Diagnostic biomarker, Kallikrein-related peptidase, *KLK10*, Prospective study, Quantitative real-time PCR

## Abstract

**Background:**

The most common malignancy in children is acute lymphoblastic leukemia (ALL). This study aimed to explore *KLK10* mRNA expression as a potential diagnostic biomarker for ALL in children and to examine the effect of chemotherapy on *KLK10* mRNA expression following the induction and after three months of receiving chemotherapy.

**Methods:**

In this prospective study, total RNA was extracted from blood samples of 23 pediatric ALL patients on diagnosis, after one month and three months of receiving chemotherapy. Healthy pediatric volunteers (*n* = 12) were selected as control individuals. After cDNA synthesis, *KLK10* mRNA gene expression levels were quantified using quantitative real-time PCR (qRT-PCR).

**Results:**

*KLK10* mRNA expression levels were significantly decreased in leukemic cells compared to their levels in cells of normal blood samples (*p* = 0.0001). *KLK10* expression levels in ALL patients after one month and three months of receiving chemotherapy decreased compared to normal blood samples (*p* < 0.0001 and *p* = 0.0175 respectively). The expression level of *KLK10* mRNA in ALL patients after one month of chemotherapy was decreased compared to their level on diagnosis (*p* = 0.4413). *KLK10* mRNA expression levels in ALL patients after three months of chemotherapy were increased compared to their level on diagnosis (*p* = 0.0602). The ROC curve illustrated that *KLK10* mRNA expression could very efficiently discriminate ALL patients from normal counterparts (AUC=0.886, 95% CI [0.7720–1.000], SE = 0.0582, *p* = 0.0004).

**Conclusion:**

*KLK10* mRNA expression could serve as a potential diagnostic molecular biomarker for ALL in children.

## Introduction

Acute lymphoblastic leukemia (ALL) is the most common pediatric malignancy (Siegel, Miller & Jemal, 2016). In the United States, 80% of diagnosed ALL cases are in children (Terwilliger & Abdul-Hay, 2017). According to a local study, the leukemia incidence rate per 100,000 pediatric age population was 3.57 in boys and 2.97 in girls (Khoshnaw, Mohammed & Abdullah, 2015). Most childhood ALL cases are developed as a result of the monoclonal proliferation of B-cell precursors (80%), mature B-cell (5%), and T-cell ALL (15%) (Kato, 2019). The onset of children’s ALL signs and symptoms are mostly related to pathological processes involved in the bone marrow and other organs due to excessive infiltration by blasts (Bernbeck et al., 2009).

Childhood ALL treatment protocols generally consist of three main phases: induction, consolidation, and maintenance, including the central nervous system prophylaxis therapy and intensive supportive care. The protocols include an intensive combination of chemotherapy regimens, and it may be supplemented with hematopoietic stem cell transplantation and/or radiation therapy. The backbone of the induction regimen typically includes vincristine, asparaginase, and a glucocorticoid (Bassan & Hoelzer, 2011). The Berlin-Frankfurt-Munich (BFM) protocol is the most extensively used consolidation scheme. This phase of therapy for standard-risk patients comprises the administration of cyclophosphamide, cytarabine, 6-mercaptopurine, and methotrexate. Additional asparaginase and vincristine regimens are used in the treatment of high-risk individuals (Brown et al., 2020; Seibel et al., 2008). In the maintenance therapy, the patients receive oral 6-mercaptopurine and methotrexate for two to three years (Jost et al., 2020). These treatment periods are based on early therapy outcomes, the intensity of the protocol, and the analysis of prognostic factors (Inaba & Mullighan, 2020; Pui et al., 2014).

Human kallikrein related-peptidases (KLKs) consist of a family of fifteen homologous secreted serine proteases, representing the largest uninterrupted cluster in the human genome. They are coded for by a family of fifteen (*KLK1-KLK15*) functional genes clustered contiguously on chromosome 19 located at q13.3-13.4 (Yousef, Luo & Diamandislz, 1999). KLKs has diverse expression profile and are found as bioactive components in many tissues and biological fluids that play essential roles in regulating normal physiological functions. The aberrant expression of several kallikrein-related peptidases has been related to various diseases and malignancies (Stefanini et al., 2015). Therefore, KLKs have played a crucial role as biomarkers. Prostate-specific antigen (PSA/KLK3) screening assay is the best example of the well-known clinical utilities of the family (Moradi et al., 2019).

KLK10 represents a potential tumor suppressor, and its mRNA and protein expressions are downregulated in ALL and breast and prostate cancers (Roman-Gomez et al., 2004; Kioulafa et al., 2009; Olkhov-Mitsel et al., 2012). Exposure to depleted uranium, as well as massive use of chemical weapons by the former Iraqi regime and recently by ISIS against the Iraqi population, have been connected to an increase in leukemia rates in various Iraqi cities (Alwan & Kerr, 2018). Our grasp of how KLK functions at the molecular level is still inadequate, and there have been few investigations on the relationship between KLK family members and hematological malignancies. Thus, this study aims to explore *KLK10* mRNA expression as a potential diagnostic biomarker for ALL and examine the effect of chemotherapy on *KLK10* mRNA expression following the induction and after three months of receiving chemotherapy.

## Materials and Methods

### Study population

This study was a prospective, analytical, observational, case-control study. The population involved in the study was pediatric individuals aged 1–15 years, including 23 patients who were newly diagnosed with ALL, and admitted to the Pediatric Department in Hiwa Cancer Hospital, Sulaimaniyah, Iraqi Kurdistan Region. Also, 12 healthy pediatric volunteers were selected as control individuals. Diagnosis of the ALL cases was established by bone marrow examination and cell immunophenotyping. Childhood ALL patients were treated according to UKALL 2019 Interim Guidelines. The Research Ethics Committee approved our research protocol at the College of Medicine, University of Sulaimani (approval number: 55). All of the recruited individuals were asked to sign a written informed consent to provide peripheral blood samples for research purposes. Blood samples were collected from leukemic patients at three different times: when diagnosed with ALL, after one month, and after three months of receiving chemotherapy.

### RNA extraction and reverse transcription

Following the manufacturer’s instructions, total RNA was extracted using a Prime Prep^TM^ Blood RNA Extraction Kit (Genet Bio, Daejeon, South Korea). The concentration and purity of RNA were evaluated by Eppendorf Biophotometer at 260 and 280 nm, also by agarose gel electrophoresis. According to the manufacturer’s instructions, the first-strand cDNA synthesis was performed using 2X SuPrimeScript RT Premix (SR-3000) kit. Conventional RT-PCR was carried out to check the quality of cDNA samples using *ACTB* primers and OnePCR™ Ultra kit (GeneDireX, Inc., Taoyuan, Taiwan). Amplification of the target sequence was affirmed using 1.5% agarose gel electrophoresis. A UV transilluminator visualized the electrophoresis bands in the gel with a gel documentation system.

### Primers

Specific primers were obtained from published articles for *KLK10* (Alexopoulou, Papadopoulos & Scorilas, 2013), *GAPDH* (Konstantoudakis et al., 2010), and *ACTB* (Shorter et al., 2008) (Table 1). The NCBI BLAST program (http://www.ncbi.nlm.nih.gov/) was used to double-check the primer sets against the human gene sequence. Sequences were also assessed to check for self-complementary or self-dimerization/hairpin using an online tool (http://www.basic.northwestern.edu/biotools/OligoCalc.html).

**Table 1:** Primers used in PCR amplifications.

**Gene**	**NCBI reference sequence**	**Primer sequence forward/reverse (5′–3′)**	**Product size bp**
*KLK10*	NM_001077500.1	F: TCTACCCTGGCGTGGTCACC	148
		R: GCAGAGCCACAGGGGTAAACAC	
*GAPDH*	NM_001289745.2	F: ATGGGGAAGGTGAAGGTCG	107
		R: GGGTCATTGATGGCAACAATATC	
*ACTB*	NM_001101	F: ATCTGGCACCACACCTTCTACAATGAGCTGCG	837
		R: CTCATACTCCTGCTTGCTGATCCACATCTGC	

### Quantitative real-time PCR (qRT-PCR)

Relative quantification of *KLK10* mRNA expression was carried out using Rotor-Gene SYBR Green RT-PCR Master Mix (Qiagen, Hilden, Germany) and a Qiagen real-time cycler (Rotor-Gene Q) following the manufacturer’s instructions. The Rotor-Gene Q cycling conditions were as follows: one initial activation step at 95 °C for 5 min, followed by 40 cycles of denaturation at 95 °C for 5 s, and a combined annealing/ extension step at 60 °C for 10 s. To evaluate data reproducibility all qPCR reactions were performed twice. Furthermore, melting curve analysis was performed for the qPCR products to verify the reaction specificity of target gene amplification; their specificity was also checked by 1.5% agarose gel electrophoresis. The calculations were made based on the comparative C_T_ (2^−ΔΔCt^) method (Schmittgen & Livak, 2008).

### Statistical analysis

The distributions of *KLK10* mRNA expression levels in ALL patients and normal controls were not Gaussian, therefore, the differences between the groups were analyzed using an appropriate non-parametric test. The Kruskal-Wallis test was used to compare the *KLK10* mRNA expression of all study groups. The Mann-Whiney U-test was used to compare the *KLK10* mRNA expression of the normal controls and the ALL patients. The Wilcoxon Signed-Rank test was used to compare the *KLK10* mRNA expression in ALL patients before and after receiving chemotherapy. Relationships between mRNA expression levels of the *KLK10* on disease diagnosis and other continuous variables were evaluated by Spearman’s correlation analysis (*r*_*s*_). The potential diagnostic value of the *KLK10* mRNA expression was examined by receiver operating characteristic (ROC) analysis. A ROC curve was constructed by plotting the true-positive rate (sensitivity) *versus* true negative rate (1-specificity) and the optimal diagnostic cut-off point was revealed. Sensitivity refers to the percentage of cases with ALL that the test correctly diagnoses as positive, whereas specificity refers to the percentage of cases without ALL that the test correctly diagnoses as negative. Hanley and McNeil method analyzed the area under the curve (AUC). An AUC value close to 1 implies a strong diagnostic test; a curve that is close to the diagonal (AUC = 0.5) has less information content and diagnostic utility (Søreide, 2009). Logistic regression analysis was performed using the mRNA expressions of the *KLK10* on disease diagnosis as a continuous variable to predict the presence of ALL. GraphPad Prism 8 software was used to analyze the data. The level of statistical significance in all tests was outlined at a probability value <0.05 (*p < 0.05, **p < 0.01, ***p < 0.001, and ****p < 0.0001). All probabilities were two-tailed.

## Results

### Clinical characteristics of ALL patients

A total of 23 newly diagnosed pediatric ALL patients were recruited for this study. ALL patients were aged 1–15 years with a mean of 6.61 ± 0.95 and a median of 5.0 years. Males were dominated by 69.57%. The mean total WBC and lymphocytes for the patients were 10.96 × 10^6^/mL and 7.28 × 10^6^/mL, respectively. The patients’ serum LDH concentration was highly increased with a mean of 1285 IU/L ±270. The diagnosis sub-type of the patients was 78.26% B-ALL and 21.74% T-ALL. Clinical variables of the study cohort are shown in (Table 2).

**Table 2:** Clinical characteristics of pediatric ALL patients.

Total number of patients	23
Age (year; Mean ±SE)	6.61 ± 0.95
Sex (*n*, %): Male	16 (69.57%)
Female	7 (30.43%)
WBC^a^ (×10^6^/mL; Mean ± SE)	10.96 ± 2.829
Lymphocytes^b^ (×10^6^/mL; Mean ± SE)	7.278 ± 2.007
Serum LDH^c^ (IU/L; Mean ± SE)	1,285 ± 270.1
ALL sub-type (*n*, %): B-ALL	18 (78.26%)
T-ALL	5 (21.74%)

**Notes.**

These data are for the newly diagnosed ALL patients before starting chemotherapy.

Reference ranges: ^a^White Blood Cells (3.5–10.0) × 10^6^/mL; ^b^Lymphocytes count (0.5–5.0) × 10^6^/mL; ^c^Lactate Dehydrogenase (240–480) IU/L.

### Expression of *KLK10* mRNA in the cohort

**Figure 1:**
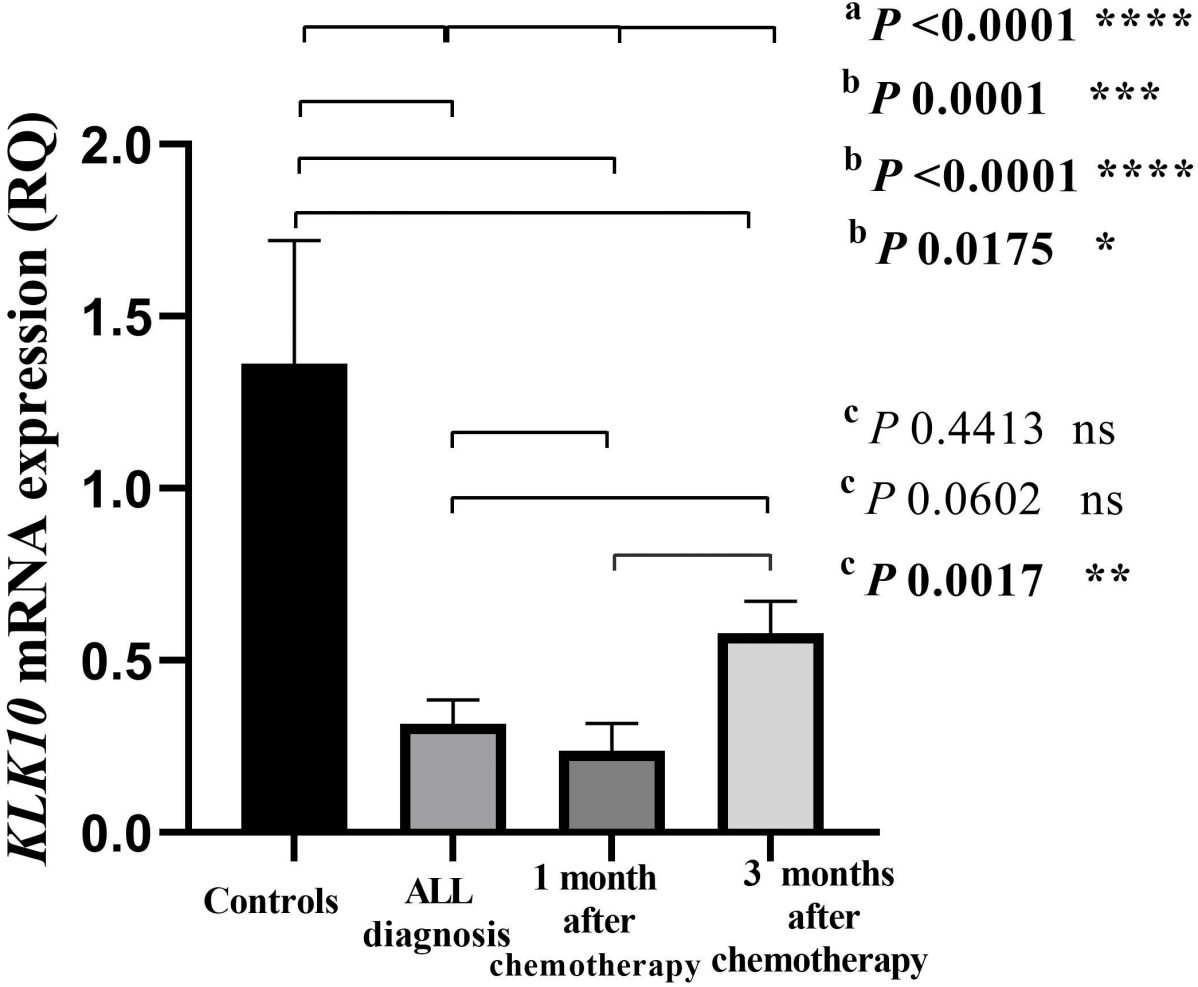
Bar-graph of *KLK10* mRNA expression in the cohort. This graph demonstrates the distribution of *KLK10* mRNA expression in each of the normal controls, the newly diagnosed ALL patients before starting chemotherapy, the patients after one month and three months of chemotherapy treatment. *P*-values were calculated using the Kruskal-Wallis test (A) to compare the *KLK10* mRNA expression of all study groups, the Mann-Whitney *U* test (B) to compare the *KLK10* mRNA expression of the normal controls and the ALL patients at the three conditions: on disease diagnosis, after one month of chemotherapy, and after three months of treatment, and the Wilcoxon Signed-Rank test (C) to compare the *KLK10* mRNA expression in the ALL patients before and after treatments. *KLK10* mRNA expression in the normal controls was significantly higher than in ALL patients at the three conditions (*p* = 0.0001; *p* < 0.0001 and *p* = 0.0175 respectively by the Mann-Whitney *U* test).

*KLK10* mRNA expression levels of all the studied groups were compared relative to each other using the Kruskal-Wallis test (*p* < 0.0001; Fig. 1). *KLK10* mRNA expression levels were significantly decreased in leukemic cells compared to their levels in cells of normal blood samples (*p* = 0.0001). The expression level of *KLK10* mRNA in ALL patients after one month and three months of receiving chemotherapy were decreased compared to their levels in normal blood samples (*p* < 0.0001 and *p* = 0.0175 respectively). The *KLK10* mRNA expression levels in ALL patients after one month of chemotherapy were decreased compared to their level in the patients on disease diagnosis (*p* = 0.4413; Fig. 1). At the same time, the *KLK10* mRNA expression level in ALL patients after three months of chemotherapy was increased compared to their level in the patients on disease diagnosis (*p* = 0.0602). The *KLK10* mRNA expression range was 0.0202–1.134 RQU with a mean of 0.316 RQU ±0.07 and a median of 0.20 RQU in the leukemic cells, while the range was 0.2934–4.629 RQU with a mean of 1.362 RQU ± 0.35, and a median of 0.96 RQU in the normal controls (Table 3). The *KLK10* mRNA expression range was 0.0056–1.496 RQU with a mean of 0.2375 RQU ±0.07, and a median of 0.08 RQU in ALL patients after one month of chemotherapy, while the range was 0.06125–1.538 RQU with a mean of 0.5793 RQU ±0.09, and a median of 0.52 RQU after three months of receiving chemotherapy. *KLK10* mRNA expression in T-ALL patients was more reduced on diagnosis and after one month of chemotherapy compared to the B-ALL patients. Its level in T-ALL patients was less increased than in B-ALL patients after receiving three months of chemotherapy (Figs. S1–S3 and Tables S1 and S2).

**Table 3:** Descriptive statistics of *KLK10* mRNA expression.

**Variables**	**Mean ± SE^b^**	**Range**	**Percentile**		
			**25th**	**Median**	**75th**
*KLK10* mRNA in normal controls (RQU^a^; *n* = 12)	1.362 ± 0.3585	0.2934–4.629	0.5126	0.9609	1.688
*KLK10* mRNA in newly diagnosed ALL patients (RQU^a^; *n* = 19)	0.316 ± 0.071	0.0202–1.134	0.0884	0.2089	0.5038
*KLK10* mRNA in ALL patients after one month of chemotherapy (RQU^a^; *n* = 19)	0.2375 ± 0.0799	0.0056–1.496	0.02337	0.08367	0.3016
*KLK10* mRNA in ALL patients after three months of chemotherapy (RQU^a^; *n* = 19)	0.5793 ± 0.0927	0.06125–1.538	0.2484	0.5288	0.7224

**Notes.**

a Relative Quantification Unit.

b Standard Error of the mean.

Another finding of this study was Spearman’s correlation coefficient of *KLK10* mRNA expression and the continuous variables in newly diagnosed ALL patients (Table 4). The expression levels were not significantly correlated with patient age, lymphocytes count, WBC, and serum LDH concentration. However, this study observed a positive correlation between WBC and ALL patients’ lymphocytes count (*r*_*s*_ = 0.83, *p* < 0.0001). A positive correlation was found between the patients’ age and serum LDH level (*r*_*s*_ = 0.71, *p* < 0.001).

**Table 4:** Correlations between *KLK10* expression and the continuous variables in newly diagnosed ALL patients.

**Variables**	***KLK10*** **mRNA** **(*n* = 19)**	**Age** **(*n* = 23)**	**Lymphocytes** **(*n* = 23)**	**WBC** ^b^ **(*n* = 23)**	**LDH** ^c^ **(*n* = 19)**
*KLK10* mRNA *r*_*s*_^a^		−0.09	−0.10	−0.31	−0.14
95% CI		−0.5339 to 0.3913	−0.5409 to 0.3830	−0.6806 to 0.1768	−0.6056 to 0.3948
*p*-value		0.71	0.68	0.19	0.6
Age *r*_*s*_^a^	−0.09		−0.48	−0.26	0.71
95% CI	−0.5339 to 0.3913		−0.7516 to −0.07408	−0.6148 to 0.1837	0.3705 to 0.8849
*p*-value	0.71		0.02*	0.23	0.001**
Lymphocytes *r*_*s*_^a^	−0.10	−0.48		0.83	−0.50
95% CI	−0.5409 to 0.3830	−0.7516 to −0.07408		0.6279 to 0.9275	−0.7830 to −0.04422
*p*-value	0.68	0.02*		<0.0001****	0.03*
WBC^b^*r*_*s*_^a^	−0.31	−0.26	0.83		−0.24
95% CI	−0.6806 to 0.1768	−0.6148 to 0.1837	0.6279 to 0.9275		−0.6345 to 0.2543
*p*-value	0.19	0.23	<0.0001****		0.32
LDH^c^*r*_*s*_^a^	−0.14	0.71	−0.50	−0.24	
95% CI	−0.6056 to 0.3948	0.3705 to 0.8849	−0.7830 to −0.04422	−0.6345 to 0.2543	
*p*-value	0.6	0.001**	0.03*	0.32	

**Notes.**

a Spearman’s Correlation Coefficient.

b White Blood Cells.

c Lactate Dehydrogenase.

The level of statistical significance in all tests was outlined at a probability value ¡0.05 (*p < 0.05, **p < 0.01, ***p < 0.001, and ****p < 0.0001).

### Diagnostic value of *KLK10* mRNA expression in ALL

**Figure 2:**
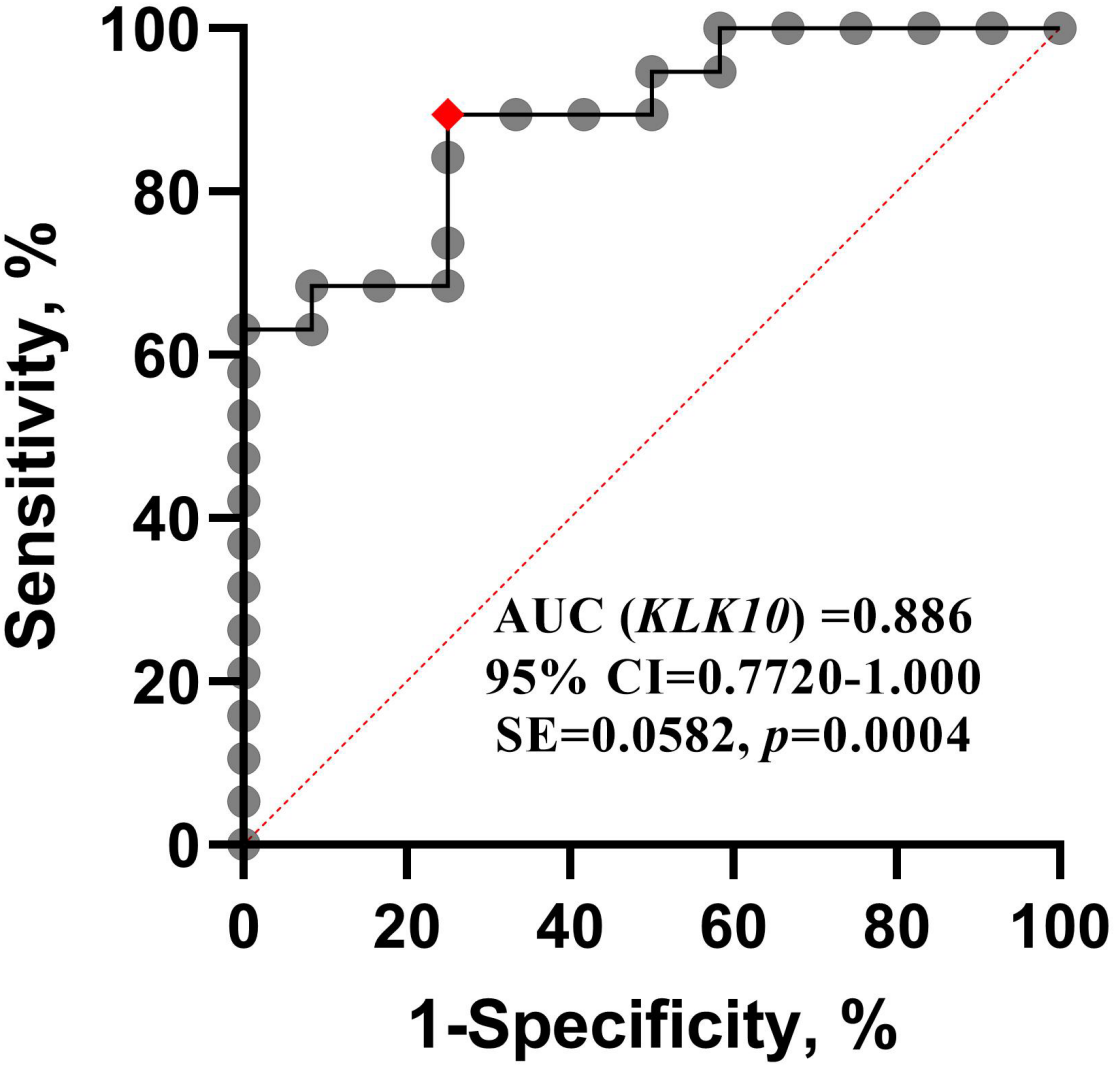
ROC curve for *KLK10* mRNA expression. Receiver operating characteristic (ROC) analysis for quantified *KLK10* mRNA expression. It shows that *KLK10* can be used successfully to diagnose ALL, and distinguish it from normal controls; AUC, Area Under Curve.

The diagnostic accuracy of the *KLK10* mRNA expression concerning ALL was evaluated by receiver operating characteristic (ROC) analysis. The area under the ROC curve (AUC) was achieved from the plotting of sensitivity *versus* (1-specificity), and the optimal diagnostic cut-off point was revealed. The ROC curve in (Fig. 2) illustrated that *KLK10* mRNA expression could very efficiently discriminate ALL from normal counterparts (AUC = 0.886, 95% CI [0.7720–1.000], SE = 0.0582, *p* = 0.0004). The ROC analysis revealed that 0.5399 RQU is the optimal diagnostic cut-off value. The sensitivity achieved with this cut-off value was 89.47%, and the specificity of the method was 75.0%.

### Logistic regression model for *KLK10* mRNA expression

To further investigate the discriminatory significance of *KLK10* mRNA expression, its quantified expression was used as a continuous variable to construct a univariate logistic regression model to predict the presence of ALL. Univariate logistic regression analysis demonstrated that patients with reduced *KLK10* mRNA expression levels establish a significant prognostic biomarker for ALL (crude odds ratio [OR] = 0.0228, 95% CI [0.0008851–0.2299], *p* < 0.0001).

## Discussion

ALL is the most prevalent hematological malignancy diagnosed in children. In ALL, precursor lymphoblasts are stopped early in their differentiation, proliferate quickly, and normal hematopoietic cells are displaced in the bone marrow (Brown et al., 2020). Despite the favorable survival rates of childhood ALL, it is essential to have sensitive and specific molecular biomarkers for the diagnosis and prognosis of the disease, assign better risk classification, and consequently better clinical results. Accumulative evidence has demonstrated that kallikrein-related peptidases have much promise in clinical oncology (Tailor et al., 2018). They serve as potential diagnostic and/or prognostic biomarkers in a variety of human cancers such as prostate (Moradi et al., 2019), breast (Figueroa et al., 2018; Watrowski et al., 2020), ovarian (Geng et al., 2017; Geng et al., 2018), colorectal (Alexopoulou, Papadopoulos & Scorilas, 2013), and gastric cancer (Liu et al., 2013). Nonetheless, KLKs are currently being researched for their function in cancer, and the investigation of other KLK family members in hematological malignancies is novel.

Roman-Gomez et al. (2004), for the first time, found that *KLK10* expression was strongly reduced at mRNA level in precursor B-cell ALL and 69% of samples diagnosed with ALL. Moreover, the study detected a loss of expression in *KLK10* due to hypermethylation in leukemic cells compared to normal cells. It was proposed as a factor for an unfavorable prognosis in childhood ALL (Roman-Gomez et al., 2004). Down-expression of *KLK10* mRNA in ALL has also been reported in other studies (Borgono, Michael & Diamandis, 2004; Paliouras, Borgono & Diamandis, 2007; Stefanini et al., 2015). Thus, the present study aimed to quantitatively analyze *KLK10* mRNA expression levels in newly diagnosed childhood ALL patients and healthy control blood donors using real-time qPCR. This study also examined the use of *KLK10* mRNA expression as a diagnostic biomarker for ALL and evaluated its level in childhood ALL patients after receiving one month and three months of chemotherapy. The choice of our sampling periods was based on common treatment periods in childhood ALL protocols.

This study found that *KLK10* mRNA expression levels were considerably lower in leukemic cells than in cells from normal blood samples. Most importantly, ROC curve analysis illustrated that *KLK10* mRNA expression could very efficiently distinguish ALL from normal counterparts, and proved the ability to differentiate between them. According to univariate logistic regression analysis patients with downregulated *KLK10* mRNA expression are more likely to develop ALL. This establishes that reduction in *KLK10* mRNA expression could be a considerable prognostic marker for ALL.

Another outcome of this investigation was Spearman’s correlation coefficient of *KLK10* mRNA expression and the continuous variables in newly diagnosed pediatric ALL patients. The levels of expression were unrelated to the patient’s age, lymphocyte count, WBC, and serum LDH concentration. Nevertheless, these findings revealed a correlation between WBC and lymphocyte count. Increases in lymphocytes are common in ALL patients (Riley & Rupert, 2015). Lactate dehydrogenase also had a positive correlation with age. This is consistent with earlier research on adults (Walaa Fikry, 2017).

In 1996, the *KLK10* was characterized as a possible tumor suppressor gene, and its expression was reduced in a breast cancer cell line (Liu et al., 1996). Further evidence of the decrease of *KLK10* mRNA expression in breast cancer tissues was discovered by *in situ* hybridization analysis (Dhar et al., 2001). This downregulation was proposed due to *KLK10* exon-3 methylation (Li et al., 2001). *KLK10* is associated with four CpG islands; the largest one is located on exon 3 of the gene (Pampalakis, Diamandis & Sotiropoulou, 2006). The downregulation of *KLK10* at the mRNA and protein levels was associated with CpG island hypermethylation (Kontos et al., 2013). It was examined that hypermethylation of *KLK10* CpG island functions a crucially significant role in tumor-specific loss and downregulation of *KLK10* mRNA and protein expressions in ALL, breast, and prostate cancers (Olkhov-Mitsel et al., 2012; Roman-Gomez et al., 2004; Sidiropoulos et al., 2005). It was reported that *KLK10* overexpression has the potential to function as a diagnostic and prognostic biomarker for pancreatic and colorectal cancers (Petraki et al., 2012; Yousef et al., 2004). Overexpression of *KLK10* mRNA was found to be an independent biomarker to predict a poor prognosis in gastric cancer, also urinary KLK10 protein played as a non-invasive biomarker to predict inoperable and incurable gastric cancer (Jiao et al., 2013; Shimura et al., 2017).

Treatment of pediatric ALL patients has been shown vast progress over the past decades, increasing considerations of remission rates and prognosis of the patients. Nevertheless, regardless of presenting favorable clinical characteristics, some patients may be overtreated or suffer from unpleasant outcomes (Smith et al., 2018; Teachey & Hunger, 2013). Therefore, discovering new prognostic biomarkers is essential to predict the patients’ outcomes and monitor their response to therapy. Kallikrein-related peptidases have emerged as key molecular biomarkers in a variety of human cancers, including ALL. The present study also performed the real-time PCR quantitative analysis of the relative expression of *KLK10* at the mRNA level for ALL patients after one month and three months of receiving chemotherapy. This is the first time *KLK10* mRNA expression in pediatric ALL patient samples has been investigated following induction therapy and three months of receiving chemotherapy. This study found that *KLK10* mRNA expression levels were significantly downregulated in ALL patients after one month and three months of receiving chemotherapy compared to their levels in normal blood samples. The expression level of *KLK10* mRNA in ALL patients after one month of chemotherapy was slightly downregulated compared to their level in the patients on disease diagnosis. At the same time, the *KLK10* mRNA expression level in ALL patients after three months of chemotherapy was upregulated compared to their level in the patients on disease diagnosis. T-ALL patients’ *KLK10* mRNA expression was lower on diagnosis, and after one month and after three months of receiving chemotherapy as compared to the B-ALL patients. However, only four T-ALL patients were considered in this research. This subtype is less prevalent, and it affects adults more than children (Bardelli et al., 2021).

ALL consists of different subtypes based on molecular alterations such as aneuploidy, chromosomal rearrangements, DNA copy number variations, and sequence mutations (Lejman et al., 2022). Chromosomal alterations such as high hyperdiploidy and t(12;21)/*ETV6-RUNX1* are well-known diagnostic and prognostic biomarkers in ALL. Also, the presence of the trisomies (+4, +10, +17, and +18) have emerged as clinically meaningful biomarkers within specific therapy regimes, although they have not yet been established as relevant biomarkers (Moorman, 2016). CD34 and CD38 proteins are detected in the majority of B-ALL patients that could be used as prognostic biomarkers. However, CD34 and CD38 expressions in certain cases were absent (Jiang et al., 2016). The primary method of leukemia cell diagnosis is flow cytometry minimal residual disease (MRD), which is reasonably rapid and affordable. MRD is an invasive and uncomfortable procedure for the patient because it demands bone marrow aspiration. Furthermore, the minimum cell prerequisite (≥2 × 10^6^) is considered to achieve a sensitivity of roughly 10^−4^ (Van Dongen et al., 2015). To help in the enhancement of prognosis before and throughout the phases of treatment, new and creative biomarkers are required. As a consequence, our findings suggest that *KLK10* mRNA expression has a favorable diagnostic utility in ALL. Downregulation in the *KLK10* expression profile following one month of chemotherapy and upregulation after three months of receiving chemotherapy might indicate that the patients were responding to treatment and the *KLK10* expression profile might have an influence on disease outcome and could be therapeutically targeted.

## Conclusion

The findings of this study demonstrate that *KLK10* mRNA expression is considerably downregulated in pediatric ALL patients compared to the control group. *KLK10* mRNA expression can be employed as a molecular biomarker in the diagnosis and prognosis of ALL. Further study in a larger cohort of ALL patients and healthy blood donors are required to investigate their use in clinical practice to diagnose and predict the presence of ALL than existing assays. More research into the *KLK10* gene’s molecular basis could contribute to a new therapeutic target for this frequent cancer.

## Supplemental Information

10.7717/peerj.13489/supp-1 Figure S1 Bar-graph of KLK10 mRNA expression in the cohort on diagnosis This graph demonstrates the distribution of *KLK10* mRNA expression in each of the normal controls, the newly diagnosed ALL patients before starting chemotherapy, and their sub-types. ^a^*P*-values were calculated using the Mann-Whitney *U* test to compare the *KLK10* mRNA expression of the normal controls and the patients.

10.7717/peerj.13489/supp-2 Figure S2 Bar-graph of *KLK10* mRNA expression in the cohort after one month of chemotherapy This graph demonstrates the distribution of *KLK10* mRNA expression in each of the normal controls, the ALL patients, and their sub-types after one month of receiving chemotherapy. ^a^*P*-values were calculated using the Mann-Whitney *U* test to compare the *KLK10* mRNA expression of the normal controls and the patients.

10.7717/peerj.13489/supp-3 Figure S3 Bar-graph of *KLK10* mRNA expression in the cohort after three months of chemotherapy This graph demonstrates the distribution of *KLK10* mRNA expression in each of the normal controls, the ALL patients, and their sub-types after three months of receiving chemotherapy. ^a^*P*-values were calculated using the Mann-Whitney *U* test to compare the *KLK10* mRNA expression of the normal controls and the patients.

10.7717/peerj.13489/supp-4 Table S1 *KLK10* mRNA expression analysis in B-ALL patients and normal controls

10.7717/peerj.13489/supp-5 Table S2 *KLK10* mRNA expression analysis in T-ALL patients and normal controls

10.7717/peerj.13489/supp-6 Supplemental Information 6 Raw data and statistical analysis of KLK10 mRNA expression in the cohort for preparation of Figure 1, Figure 2, Table 2, Table 3, and Table 4

10.7717/peerj.13489/supp-7 Supplemental Information 7 Raw data and statistical analysis of KLK10 mRNA expression in the cohort for preparation of Figure S1, Figure S2, Figure S3, Table S1, and Table S2

## Additional Information and Declarations

### Data Availability

The following information was supplied regarding data availability: The raw data is available in the Supplemental Files.
