# Supplementary figures and images for: Prospective quantitative gene expression analysis of kallikrein-related peptidase KLK10 as a diagnostic biomarker for childhood acute lymphoblastic leukemia
Source: PeerJ. 2022 May 31;10:e13489. doi: 10.7717/peerj.13489 (PMC9165590; doi:10.7717/peerj.13489)

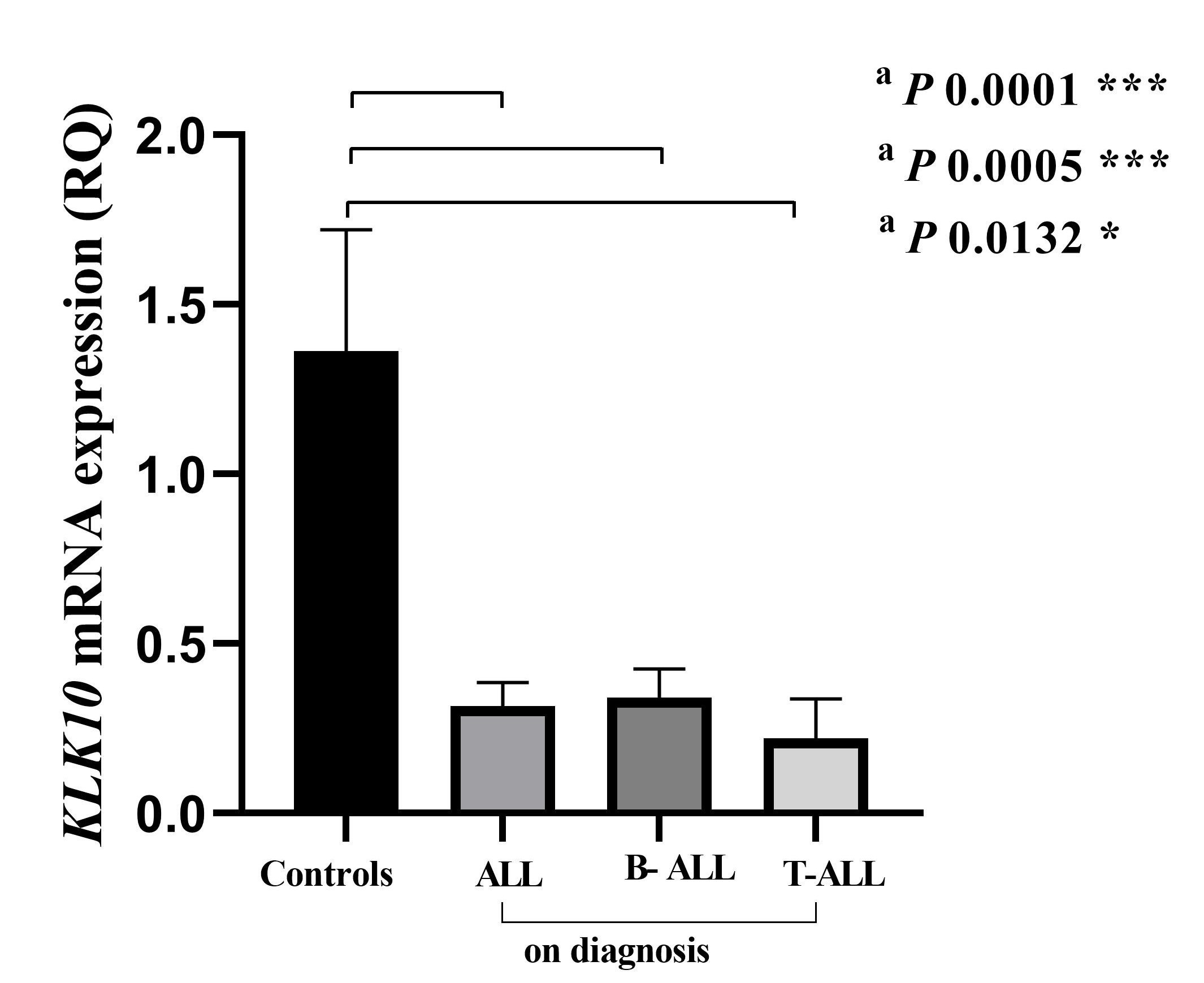

Supplement: Figure S1 — This graph demonstrates the distribution of KLK10 mRNA expression in each of the normal controls, the newly diagnosed ALL patients before starting chemotherapy, and their sub-types. aP-values were calculated using the Mann-Whitney U test to compare the KLK10 mRNA expression of the normal controls and the patients. [file peerj-10-13489-s001.jpg]

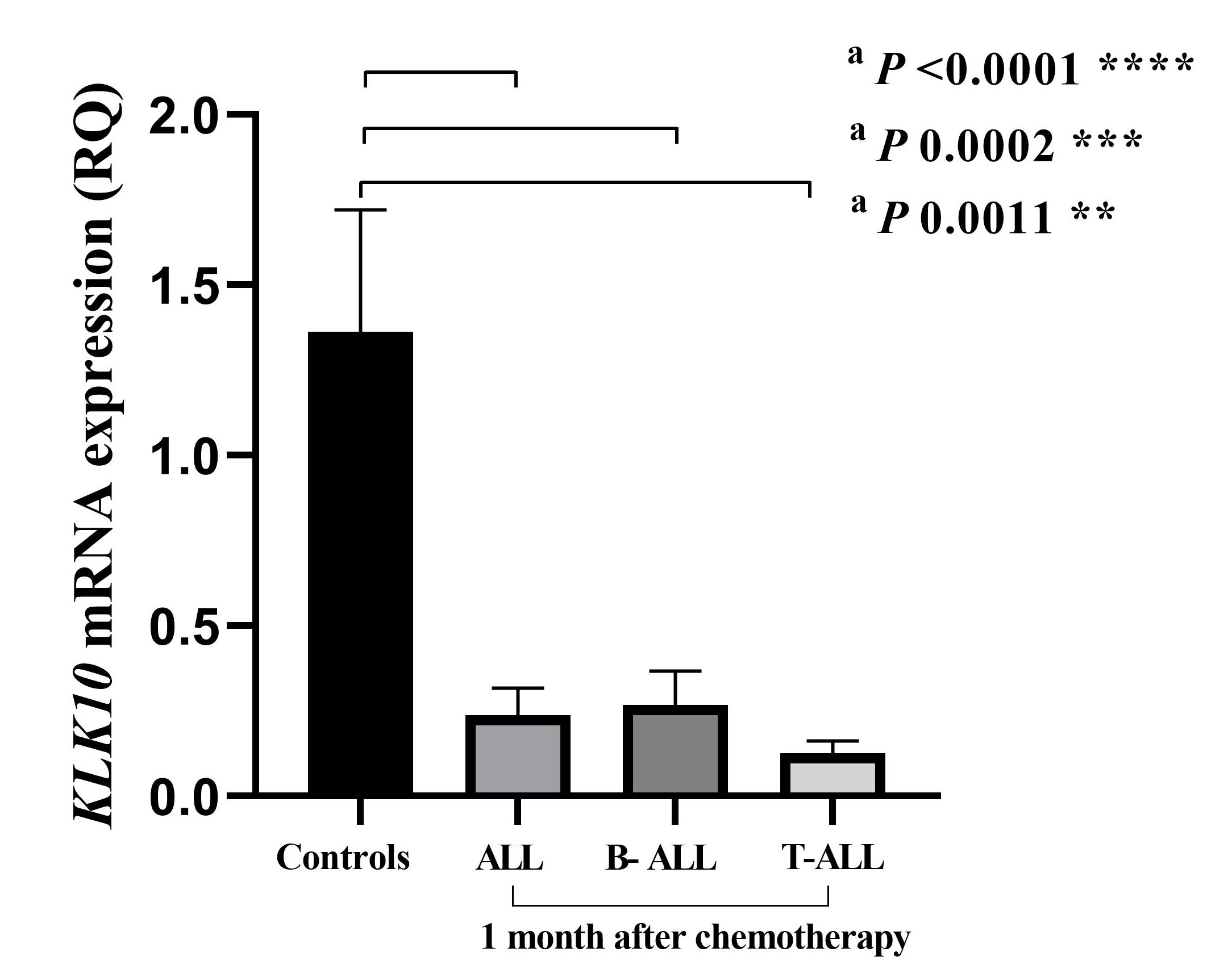

Supplement: Figure S2 — This graph demonstrates the distribution of KLK10 mRNA expression in each of the normal controls, the ALL patients, and their sub-types after one month of receiving chemotherapy. aP-values were calculated using the Mann-Whitney U test to compare the KLK10 mRNA expression of the normal controls and the patients. [file peerj-10-13489-s002.jpg]

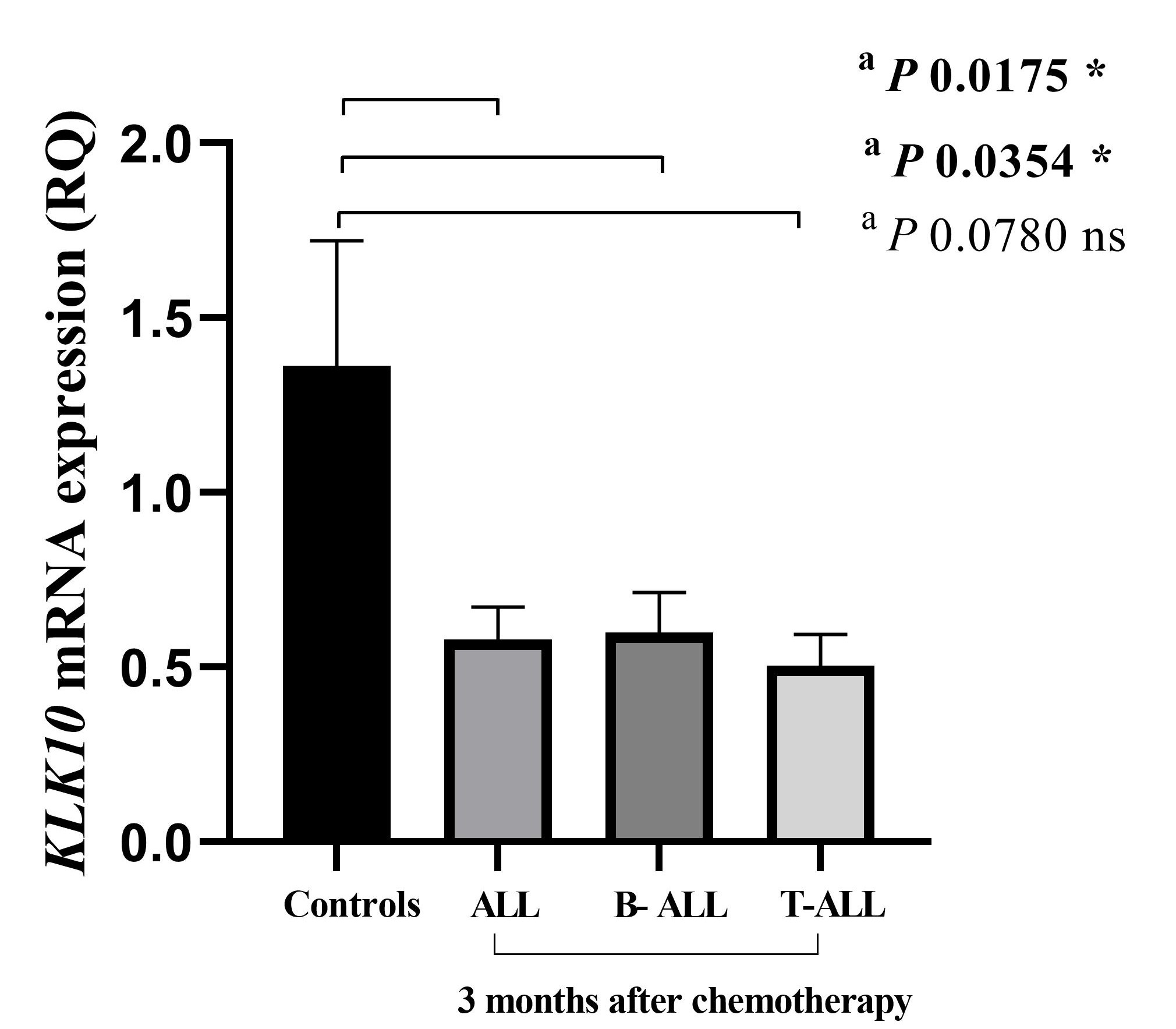

Supplement: Figure S3 — This graph demonstrates the distribution of KLK10 mRNA expression in each of the normal controls, the ALL patients, and their sub-types after three months of receiving chemotherapy. aP-values were calculated using the Mann-Whitney U test to compare the KLK10 mRNA expression of the normal controls and the patients. [file peerj-10-13489-s003.jpg]
