# Supplementary material for: Prospective quantitative gene expression analysis of kallikrein-related peptidase KLK10 as a diagnostic biomarker for childhood acute lymphoblastic leukemia
Source: PeerJ. 2022 May 31;10:e13489. doi: 10.7717/peerj.13489 (PMC9165590; doi:10.7717/peerj.13489)
Supplement: Table S1 [file peerj-10-13489-s004.docx]

| **Supplemental Table S1 *KLK10* mRNA expression analysis in B-ALL patients and normal controls.** | | | | | |
| --- | --- | --- | --- | --- | --- |
| **Variables** | **Mean± SE^b^** | **Range** | **Percentile** | | |
|  |  |  | **25th** | **Median** | **75th** |
| ***KLK10* mRNA expression (RQU^a^)** |  |  |  |  |  |
| **in normal controls (n=12)** | 1.362±0.3585 | 0.2934-4.629 | 0.5126 | 0.9609 | 1.688 |
| **in newly diagnosed B-ALL patients (n=15)** | 0.341±0.0843 | 0.07184-1.134 | 0.0988 | 0.2089 | 0.5038 |
| **in B-ALL patients after one month of receiving chemotherapy (n=15)** | 0.2673±0.1002 | 0.0056-1.496 | 0.01885 | 0.08195 | 0.4207 |
| **in B-ALL patients after three months of receiving chemotherapy (n=15)** | 0.5992±0.1158 | 0.06125-1.538 | 0.2450 | 0.4447 | 1.103 |
| ^a^ Relative Quantification Unit; ^b^ Standard Error of the mean. | | | | | |
