# Supplementary material for: Prospective quantitative gene expression analysis of kallikrein-related peptidase KLK10 as a diagnostic biomarker for childhood acute lymphoblastic leukemia
Source: PeerJ. 2022 May 31;10:e13489. doi: 10.7717/peerj.13489 (PMC9165590; doi:10.7717/peerj.13489)
Supplement: Table S2 [file peerj-10-13489-s005.docx]

| **Supplemental Table S2 *KLK10* mRNA expression analysis in T-ALL patients and normal controls.** | | | | | |
| --- | --- | --- | --- | --- | --- |
| **Variables** | **Mean± SE^b^** | **Range** | **Percentile** | | |
|  |  |  | **25th** | **Median** | **75th** |
| ***KLK10* mRNA expression (RQU^a^)** |  |  |  |  |  |
| **in normal controls (n=12)** | 1.362±0.3585 | 0.2934-4.629 | 0.5126 | 0.9609 | 1.688 |
| **in newly diagnosed T-ALL patients (n=4)** | 0.2205±0.1174 | 0.02021-0.5038 | 0.02383 | 0.1790 | 0.4586 |
| **in T-ALL patients after one month of receiving chemotherapy (n=4)** | 0.1254±0.03672 | 0.07036-0.2318 | 0.07369 | 0.09978 | 0.2028 |
| **in T-ALL patients after three months of receiving chemotherapy (n=4)** | 0.5048±0.08902 | 0.2484-0.6510 | 0.3185 | 0.5598 | 0.6360 |
| ^a^ Relative Quantification Unit; ^b^ Standard Error of the mean. | | | | | |
